# Spatial distribution and subtype‐specific expression patterns of Nectin‐4 in muscle‐invasive bladder cancer

**DOI:** 10.1111/bju.16643

**Published:** 2025-01-13

**Authors:** Csilla Olah, Lara Sichward, Boris Hadaschik, Christopher Darr, Viktor Grünwald, Ulrich Krafft, Barbara T. Grünwald, Osama Mahmoud, Mulham Al‐Nader, Peter Nyirady, Henning Reis, Tibor Szarvas

**Affiliations:** ^1^ Department of Urology University of Duisburg‐Essen Essen Germany; ^2^ Institute of Pathology, University Medicine Essen University of Duisburg‐Essen Essen Germany; ^3^ Department of Medical Oncology University of Duisburg‐Essen Essen Germany; ^4^ Dr. Senckenberg Institute of Pathology, University Hospital Frankfurt Goethe University Frankfurt Frankfurt am Main Germany; ^5^ Department of Urology, Qena Faculty of Medicine South Valley University Qena Egypt; ^6^ Department of Urology Semmelweis University Budapest Hungary; ^7^ Princess Margaret Cancer Centre Toronto Ontario Canada

**Keywords:** bladder cancer, nectin‐4, enfortumab vedotin, molecular subtype, cisplatin, immune checkpoint inhibitor, immunohistochemistry

## Abstract

**Objective:**

To investigate the expression patterns of Nectin‐4, the target molecule of the antibody‐drug conjugate enfortumab vedotin (EV), in relation to histological and molecular subtypes of urothelial bladder cancer (UBC).

**Patients and Methods:**

We assessed the protein expression patterns of Nectin‐4 in a spatially organised tissue microarray containing 1386 tissue cores from 314 consecutive patients with UBC who underwent radical cystectomy (2005–2018). Results were correlated with clinicopathological and follow‐up data, as well as with different spatial locations (tumour central vs tumour‐normal interface and primary tumour vs lymph node [LN] metastases). Additionally, we correlated Nectin‐4 expression levels with histological and molecular subtypes. Finally, we assessed the value of Nectin‐4 expression for predicting the efficacy of platinum therapy in the peri‐operative setting.

**Results:**

Nectin‐4 expression was observed in 63% of primary tumours and 87% of LN metastases, with significantly higher levels in LNs. Of the histological subtypes, the micropapillary (58%) and pure urothelial histologies (30%) were associated with the highest Nectin‐4 positivity, while the sarcomatoid (17%), squamous (15%) and small/cell‐neuroendocrine (0%) subtypes exhibited the lowest. Nectin‐4 immunopositivity rates were significantly higher in luminal (urothelial‐like [42%] and genomically unstable [34%] Lund subtypes) compared to the basal (5%) or mesenchymal (0%) molecular subtypes. Higher Nectin‐4 expression levels were associated with lower tumour stage but showed no association with overall survival. Finally, patients with low Nectin‐4 expression tended to derive more benefit from platinum‐based chemotherapy in both adjuvant and neoadjuvant settings (*P* < 0.001, *P* = 0.067).

**Conclusion:**

Our results revealed a low spatial heterogeneity of Nectin‐4 expression within the primary tumour. In contrast, differential Nectin‐4 expression was found in the context of histological and molecular subtypes. Nectin‐4‐expressing tumours may show varying sensitivity to both EV and platinum‐based chemotherapy.

**Abbreviations**
BA/SQ: basal/squamous
CC: clear cell
EV: enfortumab vedotin
FGFR: fibroblast growth factor receptor
GL: glandular
GU: genomically unstable
HR: hazard ratio
ICI: immune‐checkpoint inhibitor
IQR: interquartile range
LELC: lymphoepithelioma‐like
LN: lymph node
Mes: mesenchymal‐like
MIBC: muscle‐invasive bladder cancer
MPUC: micropapillary urothelial carcinoma
NMIBC: non‐muscle‐invasive bladder cancer
NOS: not otherwise specified
ORR: objective response rate
OS: overall survival
PUC: plasmacytoid
RC: radical cystectomy
SARC: sarcomatoid
Sc/NE: small‐cell/neuroendocrine‐like
Sq: squamous
TC: tumour central
TNI: tumour‐normal interface
UBC: urothelial bladder cancer
Uro: urothelial‐like

## Introduction

The treatment landscape of both locally advanced and metastatic urothelial bladder cancer (UBC) has largely improved in the last few years due to the approval of new drugs with different mechanisms of action including immune‐checkpoint inhibitors (ICIs), and anti‐fibroblast growth factor receptor (FGFR)‐, TROP2‐ or Nectin‐4‐targeting agents.

Enfortumab vedotin (EV) is an antibody‐drug conjugate that selectively binds to the transmembrane protein Nectin‐4, which is highly expressed in various cancers, including UBC. Upon binding and internalisation into the Nectin‐4‐expressing cells, EV delivers a potent microtubule‐disrupting agent called monomethyl auristatin E (MMAE) and induces apoptotic cell death. In the phase I EV‐101 study, which enrolled metastatic urothelial carcinoma patients with Nectin‐4‐positive tumours, who had previously received ICI therapy, EV demonstrated a superior objective response rate (ORR) of 43% [1]. In the phase III EV‐301 trial in patients with platinum‐ and ICI‐pretreated metastatic or unresectable locally advanced urothelial carcinoma, EV outperformed second‐line chemotherapy (docetaxel, paclitaxel or vinflunine), in terms of both overall survival (OS; 13 vs 9 months) and ORR (41% vs 18%) [2]. This study led to the approval of EV in the third‐line setting. Most importantly, in the currently published phase III EV‐302 trial comparing combined EV and pembrolizumab to platinum‐based chemotherapy in first‐line treatment of patients with untreated locally advanced or metastatic urothelial carcinoma, the EV combination demonstrated significantly longer progression‐free survival (12.5 vs 6.3 months) and OS (31.5 vs 16.1 months), with a manageable safety profile [3]. Based on these results, the EV/pembrolizumab combination received approval as a first‐line treatment for locally advanced or metastatic urothelial carcinoma, replacing platinum chemotherapy after over 30 years as the primary first‐line therapy. In these clinical trials, the ORR was between 41% and 44% in the third‐line and 68% in the first‐line setting, with rates of grade ≥3 adverse events of 54% and 56%, respectively [1, 2, 3, 4]. Subsequent real‐life studies confirmed the survival benefit of EV in cohorts of patients with metastatic urothelial carcinoma [5, 6]. Further studies assessing EV in earlier disease settings such as in non‐metastatic muscle‐invasive bladder cancer (MIBC; phase III EV‐303) and in high‐risk, BCG‐unresponsive non‐muscle‐invasive bladder cancer (NMIBC) as instillation therapy (EV‐104) are currently ongoing [7, 8]. In addition, in the first‐line metastatic setting, nivolumab and gemcitabine‐cisplatin combination therapy was found to be superior to gemcitabine‐cisplatin, providing a further first‐line therapy option [9]. Considering that in current EV‐containing treatment settings, 30%–60% of patients do not respond to the therapy and adverse events are common, Nectin‐4 tissue expression as a potential predictor of EV efficacy may help to optimise clinical decision‐making.

The expression of Nectin‐4 in various tumours and its impact on patient survival outcomes is currently the subject of active research. Despite the therapeutic application of EV, only a few studies have investigated Nectin‐4 tissue expression in UBC, and little is known about the spatial distribution of Nectin‐4 within the primary tumour or between primary and metastatic sites. Another interesting yet poorly characterised aspect is the potentially different expression levels of Nectin‐4 in various histological and molecular subtypes of UBC. Therefore, the present study aimed to assess the spatial expression of Nectin‐4 within the primary tumour as well as in matching positive lymph nodes (LNs) to reveal the potential tumour heterogeneity and determine the Nectin‐4 expression patterns across various secondary histological and molecular subtypes of UBC. Finally, we assessed the the value of Nectin‐4 expression in predicting platinum chemotherapy efficay in our institutional adjuvant chemotherapy cohort as well as in an independent cohort of MIBC patients who received neoadjuvant platinum treatment.

## Patients and Methods

### Patient Cohorts

This study included 314 UBC patients who underwent radical cystectomy (RC) between 2005 and 2018 at the Department of Urology, University of Duisburg‐Essen. Inclusion criteria were availability of both formalin‐fixed and paraffin‐embedded tumour samples and clinical and follow‐up data. A tumour tissue microarray was constructed with an overall number of 1386 formalin‐fixed and paraffin‐embedded tissue cores 2 mm in size. The representative tumorous regions were selected by an experienced uropathologist (H.R.). Two cores were taken from the tumour central (TC) region, two cores from the tumour‐normal interface (TNI) region, and two cores from corresponding LN metastasis. None of the patients received neoadjuvant chemotherapy, while 52 patients were treated with adjuvant chemotherapy within 90 days after RC. In addition, for validation purposes, two previously published patient cohorts with available transcriptome data and molecular subtype information were used: one cohort of patients with MIBC who were treated with neoadjuvant chemotherapy (GSE169455, *n* = 124) and another cohort of patients with MIBC who underwent RC treatment without peri‐operative chemotherapy (GSE83586, *n* = 161). In the neoadjuvant chemotherapy cohort, the molecular subtypes were determined using the transurethral resection specimens [10, 11]. The study was conducted in accordance with the Declaration of Helsinki and was approved by our institutional ethics committee (15‐6400‐BO).

### Immunohistochemistry

Nectin‐4 immunohistochemical staining was performed and evaluated exactly as previously described [12], with the only difference being the use of a Ventana Benchmark Ultra Plus device (Ventana Medical Systems, Oro Valley, AZ, USA). Nectin‐4 staining was performed on 4‐μm thick tissue microarray sections using a Nectin‐4 monoclonal antibody (anti‐Nectin‐4 antibody, clone: EPR15613‐68, Abcam, Cambridge, UK; dilution: 1:100, incubation: 32 min/37°C, antigen retrieval: CC1, incubation: 64 min/91°C) on a Ventana Benchmark Ultra Plus device (Ventana Medical Systems) according to the manufacturer's instructions. Membranous Nectin‐4 expression was classified into four groups by H.R. based on *H*‐scores: negative (0–14), weak (15–99), moderate (100–199), and strong (200–300). In subsequent analyses, the negative and weak groups were combined to form a ‘low Nectin‐4 expression’ group, while the moderate and strong groups were combined to form the ‘high Nectin‐4 expression’ group [12]. Nectin‐4 expression was separately evaluated in the TC and TNI regions, and in positive LNs. Two cores were sampled from each region, and their Nectin‐4 *H*‐scores were averaged for evaluation. For a subcohort of 173 TC samples, molecular subtype information based on the 13 immunohistochemical marker‐based Lund taxonomy classification system was available [13]. The molecular subtypes identified included urothelial‐like (Uro), genomically unstable (GU), basal/squamous (BA/SQ), mesenchymal‐like (Mes), and small‐cell/neuroendocrine‐like (Sc/NE) subtypes.

### Statistical Analysis

Nectin‐4 expression (as a continuous *H*‐score) was compared among the TC region, TNI region, and positive LNs using the Mann–Whitney test. Similarly, Nectin‐4 expression was compared among different histological and molecular subtypes. The correlation of Nectin‐4 expression (*H*‐score) with the different tumour regions was also tested using Pearson's correlation test. Correlations between clinicopathological parameters and dichotomised Nectin‐4 expression level (low vs high) were tested using either the chi‐squared test (for dichotomised clinical variables) or the Mann–Whitney test (for continuous variables). Correlations between the expression of Nectin‐4 and different molecular subtype markers were tested using Pearson's correlation test. The Kaplan–Meier log‐rank test and Cox univariable and multivariable analyses were performed to evaluate OS. The results of survival analyses were visualised using Kaplan–Meier curves and Forest plots. Statistical analyses were conducted using the SPSS software package (IBM SPSS Statistics for Windows, version 25, IBM Corp., Armonk, NY, USA) and R Studio (Version 4.2.1). *P* values ≤0.05 were taken to indicate statistical significance.

## Results

### Nectin‐4 Staining Characteristics

Nectin‐4 immunohistochemical staining was evaluated specifically by taking only the membranous staining into account as the functional requirement for EV binding, while cytoplasmic staining was not evaluated (Fig. S1). In rare cases nuclear reactivity was observed (overlay). The immunohistochemistry protocol was validated using the specific Nectin‐4 expression level in normal urothelium.

### Nectin‐4 Expression Heterogeneity Within the Primary Tumours and Between the Primary and LN Metastatic Sites

We examined Nectin‐4 expression in a consecutive institutional cohort of 314 UBC patients. Detailed patient characteristics are presented in Table 1A. Nectin‐4 staining was evaluable for 287 TC regions, 252 TNI regions and 55 positive LNs.

**Table 1:** Patients' characteristics (A) and comparison of Nectin‐4 staining among spatial localisations (B).

(A) Variables	Tissue
Total number of patients, *n*	314
Age at baseline median (range)	69.4 (37.2–90.3)
Sex, *n* (%)	
Male	242 (77)
Female	72 (23)
Cystectomy T‐stage, *n* (%)	
pT1	23 (7)
pT2	82 (26)
pT3	126 (40)
pT4	59 (19)
na	24 (8)
Positive surgical margin, *n* (%)	50 (16)
Vascular invasion, *n* (%)	51 (16)
Lymphovascular invasion, *n* (%)	121 (39)
Lymph node metastasis, *n* (%)	88 (28)
Distant metastasis, *n* (%)	17 (5)
Chemotherapy regimen (Gem/Cis), *n* (%)	52 (17)
Number of cycles, median (range)	4 (1–8)
Single (only one cycle)	4 (1)
Number of patients who died, *n* (%)	206 (66)
Follow‐up time, median (range) months	23 (1–185)
Follow‐up for survivor's median (range)	68 (1–185)

(B) Nectin‐4 immunohistochemistry	*n* (*H*‐score [IQR])
TC region	287 (40 [0–100])
Tu‐No interface	252 (40 [0–110])
Positive LNs	55 (100 [60–200])

IQR, interquartile range; LN, lymph node; TC, tumour central; Tu‐No, tumour‐normal.

**Fig. 1:**
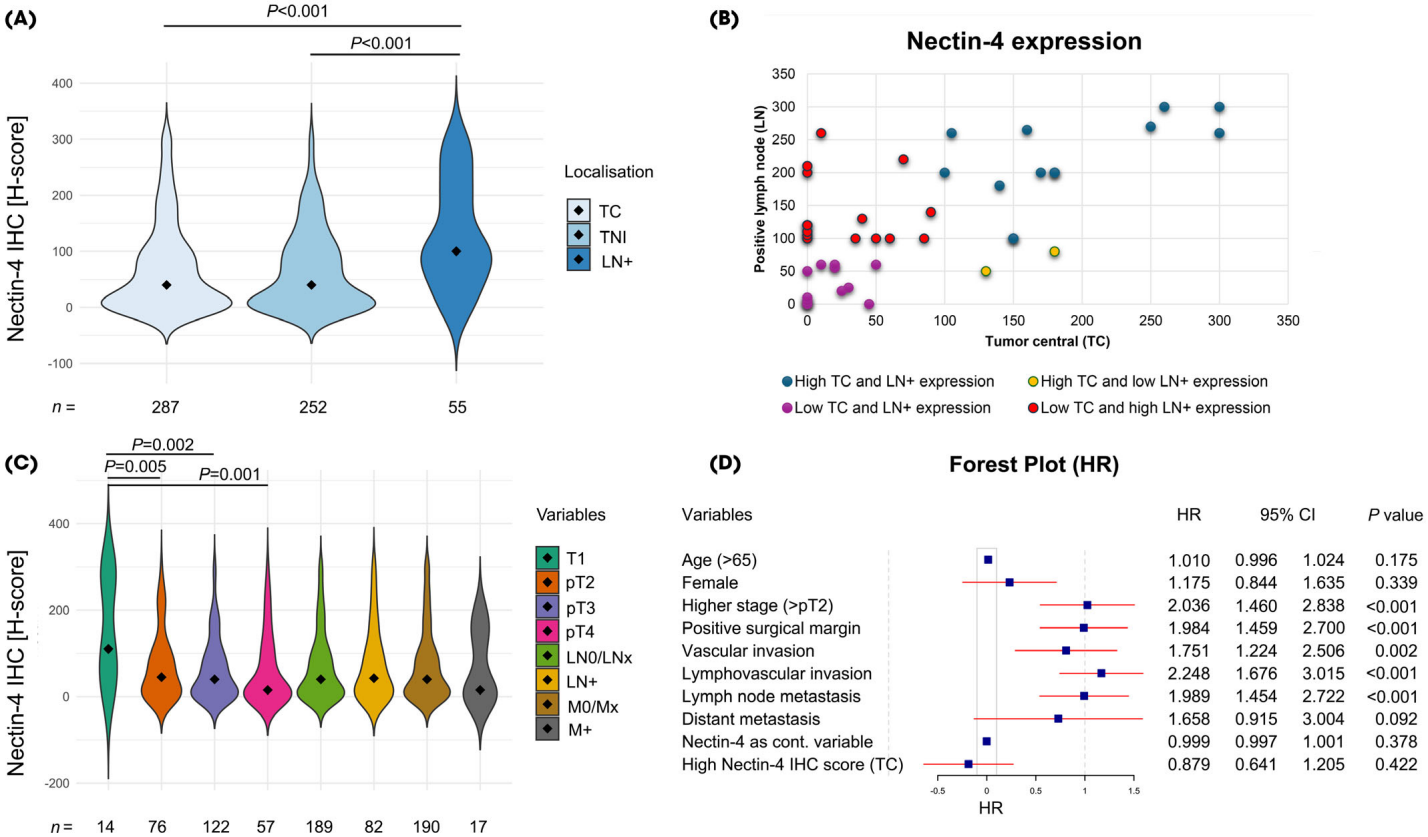
Nectin‐4 expression in different tumour localisations: (**A**) the tumour central (TC) region, (**B**) the corresponding positive lymph nodes and(**C**) at different tumour stages. (**D**) Forest plot presentation of the associations of various clinicopathological factors and Nectin‐4 expression with patients' overall survival. HR, hazard ratio; IHC, immunohistochemistry; LN+, positive lymph node; M+, presence of distant metastasis; TNI, tumour‐normal interface.

We observed low spatial heterogeneity (TC vs TNI) within the primary tumour (Pearson *r* = 0.829, *P* < 0.001), which suggests stable expression across various tumour regions. When comparing all three compartments assessed (TC region, TNI region and LNs), we found significantly higher Nectin‐4 expression in the LN metastases (median *H*‐score: 100; interquartile range [IQR]: 60–200) compared to the TC region (median *H*‐score: 40; IQR: 0–100) and TNI (median *H*‐score: 40; IQR: 0–110) regions, (Table 1B, Fig. 1A). For further analyses, we used the central tumour Nectin‐4 expression level when evaluating the results.

A total of 105 TC samples were classified as negative (37%), 99 as weak (34%), 55 as moderate (19%), and 28 (10%) as strong for Nectin‐4 expression. In contrast, only seven LN metastases were classified as negative (13%) and 15 as weak (27%), while 16 were classed as having moderate (29%) and 17 (31%) as having strong Nectin‐4 expression (Fig. S2).

For matched TC region vs corresponding LN analysis of Nectin‐4 expression levels, 51 sample pairs were available for direct comparison between the primary tumours and positive LNs. Consistently low (negative/weak) expression in both compartments (TC region and LNs) was observed in 18 patients (35%), and consistently high (moderate/strong) expression in 13 patients (25%). Low expression in the TC region but simultaneously high expression in the LNs was detected in 18 patients (35%), while high expression in the TC region but low expression in the LNs was observed in only two patients (5%; Fig. 1B). These results suggest that high Nectin‐4 expression in the primary tumour is rarely accompanied by low expression in the positive LNs, while low Nectin‐4 expression in the primary tumour is not necessarily associated with lower expression in the LN metastases.

### Association between Nectin‐4 Expression and Clinical Parameters

Nectin‐4 expression in primary tumours showed no association with patients' age, sex, LN status or distant metastasis status, or the presence of positive surgical margins, or vascular and lymphovascular invasion. Only lower tumour stage was associated with significantly higher Nectin‐4 expression (T1 vs ≥pT2; *P* = 0.005 [Fig. 1C, Table S1]). Interestingly, high Nectin‐4 expression in the LNs was significantly associated with the presence of lymphovascular invasion in primary tumours (*P* = 0.038 [Table S1]).

### Nectin‐4 Expression across Histological Subtypes

The studied cohort included only urothelial carcinomas as main histological pattern; however, additional histological subtypes were also present. Eleven samples exhibited more than one histological subtype and were excluded, leaving 276 samples for subtype‐specific analysis. In the TC region, 49% of tumours (136/276) were of the pure urothelial (not otherwise specified [NOS]) type. The most frequently observed histological subtypes were the squamous (Sq) urothelial carcinoma subtype in 22% (60/276) and micropapillary urothelial carcinoma (MPUC) in 13% (36/276). The following histological subtypes were also identified: sarcomatoid (SARC) in 4% (12/276), glandular (GL) in 4% (10/276), small‐cell/neuroendocrine (Sc/NE) in 3% (7/276), lymphoepithelioma‐like (LELC) in 2% (6/276), clear cell (CC) in 1% (4/276) and plasmacytoid in 1% (3/276). One sample exhibited giant‐cell and a further sample trophoblastic histology. Table S2 and Figure S2 present in detail the Nectin‐4 expression levels (negative, weak, moderate, strong) identified in the various histological subtype groups.

**Fig. 2:**
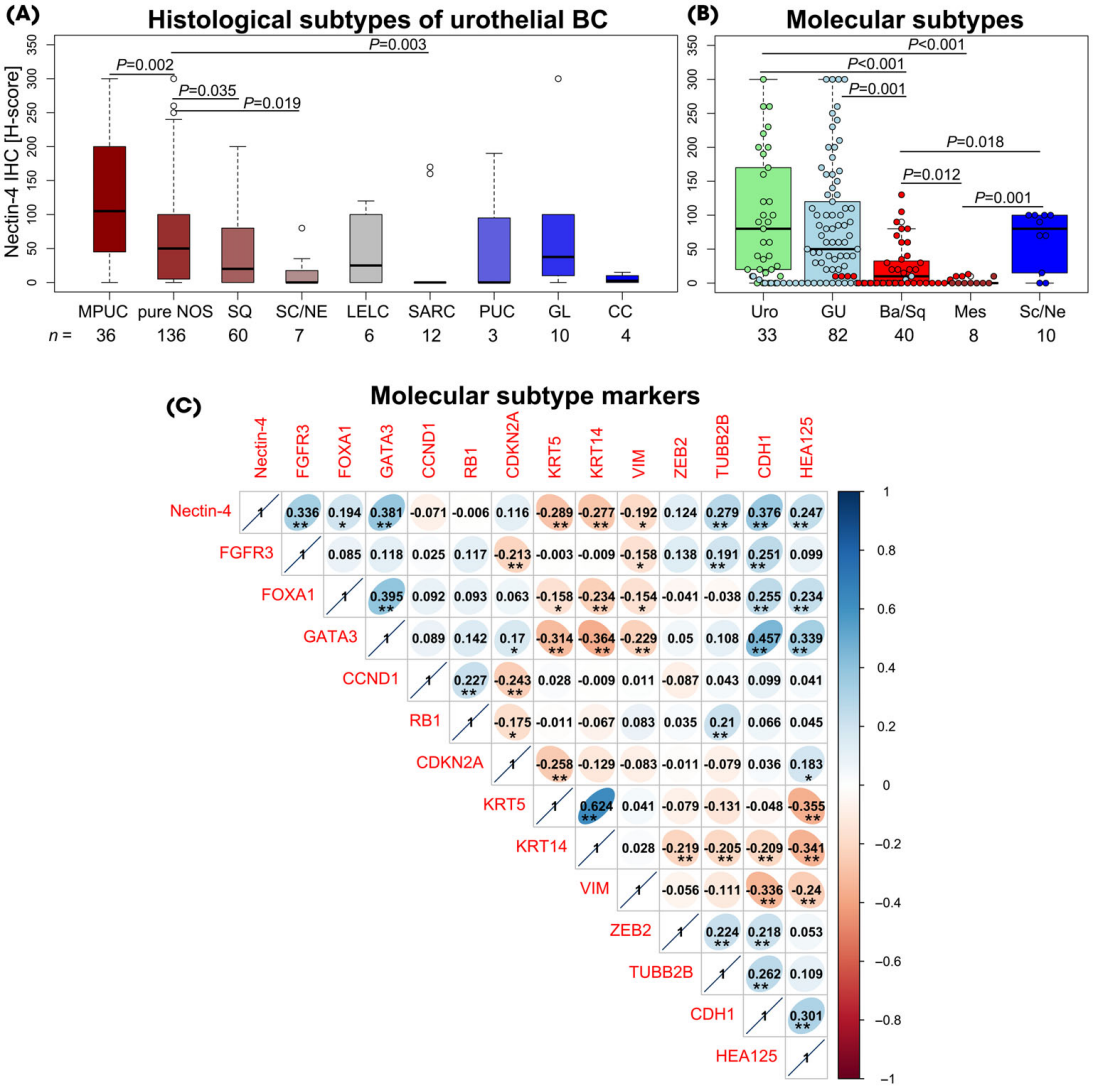
Nectin‐4 expression in different (**A**) histological subtypes and (**B**) molecular subtypes in the tumour central region. (**C**) Correlation between the expression of Nectin‐4 and 13 molecular subtype markers. Statistical significance: **P ≤ 0.01 and *P ≤ 0.05 in plot C. BA/SQ, basal/squamous; CC, clear‐cell; GL, glandular; GU, genomically unstable; IHC, immunohistochemistry; LELC, lymphoepithelioma‐like; Mes, mesenchymal‐like; MPUC, micropapillary urothelial carcinoma; NOS, urothelial carcinoma without subtype/not otherwise specified; PUC, plasmacytoid; SARC, sarcomatoid; Sc/NE, small‐cell/neuroendocrine; Sq, squamous; Uro, urothelial‐like.

Thirty percent of tumours (41/136) with pure NOS histology exhibited high Nectin‐4 expression. Significantly higher Nectin‐4 expression levels were observed in tumours with MPUC histology (*P* = 0.002), with 58% (21/36) of these tumours showing high Nectin‐4 expression. In contrast, significantly lower Nectin‐4 expression was detected in the Sq and SARC subtypes compared to pure NOS tumours, with only 15% (9/60) of Sq tumours and 17% (2/12) of SARC tumours exhibiting high Nectin‐4 expression (*P* = 0.035, 0.003). Sc/NE tumours showed only negative or weak Nectin‐4 expression (*P* = 0.019; Fig. 2A). The single cases found of giant‐cell (*H*‐score of 250) and trophoblastic histology (*H*‐score of 5) are not shown in Fig. 2A.

The cohort included 14 NMIBC tumours (9 pure NOS, 2 MPUC, 1 Sq, 1 LELC, and 1 GL) with available Nectin‐4 expression data. The exclusion of T1 tumours did not meaningfully alter the results. Significantly higher Nectin‐4 expression was observed in MPUC tumours (*P* = 0.010), whereas lower expression was found in Sc/NE and SARC variants (*P* = 0.022 and *P* = 0.019, respectively) compared to pure NOS tumours (Fig. S3A).

### Heterogenous Nectin‐4 Expression Levels across Various Molecular Subtypes

Molecular subtypes of UBC are characterised by distinct mutational landscapes and gene expression patterns, which are also reflected in differences in their prognoses and therapeutic sensitivities. In the present study, we examined whether differences in Nectin‐4 expression patterns could be identified among various molecular subtypes. Molecular subtype information according to the immunohistochemistry‐based Lund taxonomy classification system was available for 173 patients. The subtype distribution (considering the TC region) was as follows: 33 Uro (19%), 82 GU (47%), 40 BA/SQ (23%), 8 Mes (5%), and 10 Sc/NE (6%). Table S3 and Figure S2 present in detail the Nectin‐4 expression levels (negative, weak, moderate, strong) in the various molecular subtype groups.

In the primary tumour tissues, high Nectin‐4 expression levels were observed in the Uro (42%, 14/33) and GU (34%, 28/82) subtypes, which are characterised by high expression levels of the luminal genes. These expression levels were significantly higher compared to the BA/SQ (20%, 8/40) and Mes (0%, 0/8) subtypes (Fig. 2B). The exclusion of eight T1 tumours (three Uro and five GU tumours) with available Nectin‐4 expression and molecular subtype data did not affect the results (Fig. S3B).

We also examined the correlations between the expression levels of Nectin‐4 and the 13 subtype markers separately. Nectin‐4 showed a significant positive correlation with the luminal markers FGFR3, FOXA1 and GATA3, as well as the neuronal markers TUBB2B and HEA125. In contrast, Nectin‐4 negatively correlated with the basal markers KRT5 and KRT14. Finally, inconsistent correlations were found with mesenchymal markers: Nectin‐4 correlated positively with CDH1, but negatively with VIM (Fig. 2C).

### Association between Nectin‐4 Expression and Patient Survival

Cox analysis revealed higher tumour stage (*P* < 0.001), positive surgical margin status (*P* < 0.001), the presence of vascular and lymphovascular invasion (*P* = 0.002 and *P* < 0.001, respectively), and the presence LN metastasis (*P* < 0.001) to be significantly associated with poor OS (Fig. 1D). Higher tumour stage (hazard ratio [HR] 1.537, 95% CI 1.055–2.241; *P* = 0.025), and the presence of positive surgical margins (HR 1.574, 95% CI 1.086–2.281; *P* = 0.017) and lymphovascular invasion (HR 1.647, 95% CI 1.162–2.335; *P* = 0.005) proved to be independent risk factors for OS. However, Nectin‐4 expression in primary cancers in an era before availability of EV had no impact on survival (Fig. 1D).

### Platinum‐Predictive Value of Nectin‐4 Expression

A subset of patients with available Nectin‐4 expression data for the TC region (*n* = 180) had an indication for adjuvant chemotherapy based on the histological findings at RC (pT3/4 or LN+). However, only 43 patients received adjuvant chemotherapy (chemotherapy cohort), while 137 patients either refused or were ineligible for platinum therapy (non‐chemotherapy cohort). We directly compared the predictive value of Nectin‐4 expression for chemotherapy efficacy by comparing OS between the chemotherapy and non‐chemotherapy cohorts in high and low Nectin‐4 expression groups. We observed a significant OS benefit for the chemotherapy cohort in the low Nectin‐4 expression group, while in patients with high Nectin‐4 expression only an insignificant trend could be observed towards better OS (log‐rank: *P* < 0.001 vs *P* = 0.134 [Fig. S4A]). This surprising observation was further assessed in an external validation cohort with available transcriptome data, which included patients who received neoadjuvant chemotherapy and patients treated only with RC. Similarly to our initial observation, a trend for OS benefit for chemotherapy‐treated patients could only be observed in the low Nectin‐4 expression group (log‐rank: *P* = 0.067), while patients with high Nectin‐4 expression levels did not benefit from neoadjuvant platinum‐based chemotherapy (log‐rank: *P* = 0.450; Fig. S4B). Patients with high Nectin‐4 expression levels may derive less benefit from platinum‐based chemotherapy, indicating that alternative therapies, such as earlier EV therapy, may be more appropriate.

## Discussion

In the present study, we examined the heterogeneity of membranous Nectin‐4 expression patterns within primary UBC, between primary tumour and metastatic LN sites, and across secondary histological and molecular subtypes. We found low intra‐tumoral heterogeneity in the primary tumour and significantly higher Nectin‐4 expression levels in metastasis‐bearing LNs. Nectin‐4 expression was highest in the micropapillary subtype and lowest in the SARC subtype. When comparing Nectin‐4 expression levels across molecular subtypes, these were high in the Uro, GU and Sc/NE subtypes, and low in the BA/SQ and Mes subtypes. In addition, we found that high Nectin‐4 expression resulted in less benefit from peri‐operative platinum‐based therapy.

Tumoral Nectin‐4 expression is assumed to be positively associated with the efficacy of EV treatment. Therefore, in the EV‐101 and EV‐201 clinical trials, only patients with positive Nectin‐4 expression were enrolled. However, in the EV‐301 trial, the presence of Nectin‐4 expression was not set as an inclusion criterion, based on the assumption that nearly all advanced urothelial carcinoma exhibits high Nectin‐4 expression [1, 2, 4]. In contrast to this assumption, retrospective real‐world studies have reported no or low Nectin‐4 expression in a considerable number of UBC cases. A study in patients with metastatic urothelial carcinoma found no Nectin‐4 expression in 20% of patients, with a median *H*‐score of only 110 [12]. In a further cohort including only upper tract UCs, 35% of patients were Nectin‐4‐negative [14]. Consistent with these findings, we found 37% of RC‐treated patients to be Nectin‐4‐negative and a further 34% of patients exhibited only weak Nectin‐4 expression, with a median *H*‐score of 40 for the whole cohort. Furthermore, in our cohort, high Nectin‐4 expression was significantly associated with lower tumour stage, which is in accordance with a former study that found higher Nectin‐4 expression levels in NMIBC samples (with 87% staining positivity) than in MIBC samples (with only 58% staining positivity) [15]. These findings challenge the former assumption that all UBCs express high levels of Nectin‐4 and show that Nectin‐4 expression can vary among UBCs. Moreover a considerable number of MIBC patients are Nectin‐4‐negative.

Klümper et al. [12] found that high Nectin‐4 expression was associated with significantly better ORR and progression‐free survival in EV‐treated metastatic urothelial carcinoma patients (*n* = 47; both *P* < 0.001), suggesting that Nectin‐4 immunohistochemistry may help select patients who will benefit from EV treatment. Interestingly, the authors observed significantly higher Nectin‐4 expression levels in primary tumours compared to metastatic sites, which may have a further impact on therapy response rates [12].

In the present study, we examined – for the first time – the spatial heterogeneity of membranous Nectin‐4 expression within primary UBCs and between primary tumours and positive LNs. We observed low spatial heterogeneity between the central and peripheral (TNI) regions of the primary tumour. However, we found significantly higher Nectin‐4 expression in the matched positive LNs compared to the primary tumours (median *H*‐score 100 vs 40). Interestingly, high Nectin‐4 expression in the primary tumour strongly correlated with high expression in LN metastases, but Nectin‐4 positivity was also frequently seen in cases with no Nectin‐4 expression in the primary tumour. This suggests that patients with low primary tumour Nectin‐4 levels might still benefit from EV therapy. Therefore, metastasis biopsy or Nectin‐4 positron emission tomography imaging could improve future patient selection for EV therapies [16].

Our results are complementary to the former findings by Klümper et al. [12], who described significantly lower Nectin‐4 expression in therapy‐naïve distant metastases compared to corresponding primary tumour tissues (median *H*‐score 40 vs 110). Another study in patients with metastatic urothelial carcinoma who received chemotherapy and ICIs found similar membranous Nectin‐4 expression levels in primary tumours and distant metastases [17]. The differences in the above results might be explained by relatively small sample sizes, and differences in the metastatic sites investigated as we assessed only LN metastases, while the other two studies assessed biopsies from distant metastases. A study by Miyake et al. [18], which included 13 cases of urothelial MIBC with matched positive LNs, found no association in Nectin‐4 expression when comparing RC and LN specimens. This result confirms that examining Nectin‐4 expression only in the primary tumour may not adequately predict patients' EV sensitivity.

In the present work, in which we analysed the largest number of UBC patients with available histological subtype and Nectin‐4 expression data to date, we found significant differences in Nectin‐4 immunostaining among histological subtypes. MPUCs exhibited the highest Nectin‐4 expression level, with a positivity rate of 83% compared to 70% in pure urothelial carcinoma NOS. Many histological subtypes showed Nectin‐4 expression levels similar to those found in pure urothelial carcinoma NOS, while the Sq urothelial carcinomas and the SARC subtype exhibited significantly lower Nectin‐4 levels. Moreover, the Sc/NE subtypes showed only low or negative Nectin‐4 expression. Two available MIBC studies on this topic reported similar Nectin‐4 positivity rates of 80% (57/82) and 68% (15/22) in pure urothelial carcinoma NOS, when compared with our cohort, which showed a rate of 70% (95/136) [15, 19]. Based on the association shown between Nectin‐4 expression and EV sensitivity, these results indicate that some subgroups (SARC, Sc/NE) may achieve lower anti‐tumour activity when treated with EV.

The results for MPUC are conflicting in the literature. One study found a Nectin‐4 positivity rate of 79% (15/19) for MPUC, while another reports a rate of only 28% (3/11) positivity [15, 19]. Our results are consistent with the first study, as we detected an 83% positivity rate in MPUC. Both studies also found relatively high Nectin‐4 expression rates in Sq urothelial carcinoma, with rates of 80% (8/10) and 70% (7/10). Only the second study included detailed data on staining intensities, reporting that 60% of Sq urothelial carcinomas showed high expression. In our study, positive Nectin‐4 expression was found in 60% (46/60) of the Sq subtype, but only 15% (9/60) exhibited high expression. Regarding the Sc/NE subtype, the first study described a low expression rate of 21% (4/19). Similarly, the other study found no positive Nectin‐4 expression in any of the Sc/NE (0/15) urothelial carcinomas [15, 19]. An independent study further confirmed significantly lower Nectin‐4 expression in the Sc/NE variant compared to pure NOS (mean *H*‐score: 20 ± 44 vs 122 ± 79; *P* = 0.015), while no significant differences were observed between other variants and pure NOS [18]. In our study, there was also no (5/7) or weak expression (2/7) in the Sc/NE subtype. The first study also included SARC urothelial carcinoma samples and described a Nectin‐4 positivity rate of only 10% (1/10), which is similar to that found in our study – positive expression was observed in only 2/12 tumours [15]. Rodler et al. [20] analysed bladder cancer cases with different histologies and found relatively low Nectin‐4 expression levels in SARC urothelial carcinoma samples, with 67% (16/24) showing low or negative expression.

Former *in silico* analyses in seven MIBC cohorts revealed heterogenous *Nectin‐4* gene expression rates across molecular subtypes. The authors described significantly higher *Nectin‐4* expression in the luminal subtypes, and accordingly a positive correlation with typical luminal genes, such as *GATA3, FOXA1* and *PPARG* [21]. A subsequent study reported significantly higher *Nectin‐4* gene expression in tumours with consensus luminal subtypes compared to the BA/SQ subtype [22]. In accordance with these results, we observed higher Nectin‐4 tissue expression in tumours with high luminal protein expression (Uro and GU). Additionally, Nectin‐4 expression positively correlated with the luminal markers FGFR3, FOXA1 and GATA3, while it negatively correlated with the basal markers KRT5 and KRT14. These results suggest that molecular subtypes could potentially aid in the selection of patients for EV therapy in the future.

In our MIBC‐dominated institutional RC cohort, we observed no association between Nectin‐4 expression and patients' survival. Similarly, Bahlinger et al. [22] found no survival differences between patients with high Nectin‐4 expression analysing the TCGA dataset and a retrospective, EV‐untreated MIBC cohort. However, we found that cases with low Nectin‐4 expression derive a stronger OS benefit from adjuvant platinum‐based treatment, which was further confirmed in an *in silico* dataset of MIBC after neoadjuvant platinum‐based treatment. These results suggest that Nectin‐4 immunostaining may help to guide treatment: patients with lower Nectin‐4 expression levels could be recommended platinum treatment (e.g., first‐line platinum/nivolumab), while higher expression levels favour EV therapy (e.g., first‐line EV/pembrolizumab).

Our study has several limitations. First, our cohort did not include patients who received EV therapy; therefore, we could not directly assess the value of Nectin‐4 in predicting EV therapy efficacy. Furthermore, even in our comparatively large patient cohort, case numbers for rare histological or molecular subtypes remain low, thus limiting the statistical evaluability of the results.

In conclusion, assessing Nectin‐4 expression in a large number of UBC samples, we observed low spatial heterogeneity within the primary tumour, while we found significantly higher expression in LN metastases. Nectin‐4 expression showed a heterogeneous pattern in different molecular subtypes as classified according to the Lund taxonomy system, showing the highest expression in the Uro and GU subtypes. Additionally, Nectin‐4 expression was significantly higher in MPUC and significantly lower in the SARC, Sq and Sc/NE histological subtypes compared to pure urothelial carcinoma NOS subtypes. Overall, these data provide a better insight into the expression patterns of Nectin‐4 and may provide valuable information for future therapeutic decision‐making as well as for clinical trials.

## Disclosure of Interests

All authors declare no conflict of interest.

## Funding

This work was supported by the K139059 grant of the Ministry for Innovation and Technology from the source of the National Research Development and Innovation Fund. We acknowledge support from the Open Access Publication Fund of the University of Duisburg‐Essen.

## Supporting information


**Table S1.** χ^2^ correlation between Nectin‐4 expression and patients’ clinicopathological parameters in tumor central regions and positive lymph nodes.
**Table S2.** Nectin‐4 expression in different secondary histological subtype groups.
**Table S3.** Nectin‐4 expression in different molecular subtype groups.
**Fig. S1.** Histomicrophotographs of different scenarios in Nectin‐4 membranous staining.
**Fig. S2.** Nectin‐4 expression in different localization and in primary tumors with different histological subtypes.
**Fig. S3.** Nectin‐4 expression in muscle‐invasive bladder tumors across different histological subtypes (A) and molecular subtypes (B) in the tumor central region.
**Fig. S4.** Kaplan–Meier overall survival curves stratified by chemotherapy treatment (chemo vs non‐chemo) in low and high Nectin‐4 expression groups in the institutional Essen cohort (A) and external validation Lund validation cohort (B).
