# Supplementary material for: Spatial distribution and subtype‐specific expression patterns of Nectin‐4 in muscle‐invasive bladder cancer
Source: BJU Int. 2025 Jan 13;136(2):297–305. doi: 10.1111/bju.16643 (PMC12256731; doi:10.1111/bju.16643)
Supplement: Supplementary file 1 — Table S1. χ2 correlation between Nectin‐4 expression and patients’ clinicopathological parameters in tumor central regions and positive lymph nodes. Table S2. Nectin‐4 expression in different secondary histological subtype groups. Table S3. Nectin‐4 expression in different molecular subtype groups. Fig. S1. Histomicrophotographs of different scenarios in Nectin‐4 membranous staining. Fig. S2. Nectin‐4 expression in different localization and in primary tumors with different histological subtypes. Fig. S3. Nectin‐4 expression in muscle‐invasive bladder tumors across different histological subtypes (A) and molecular subtypes (B) in the tumor central region. Fig. S4. Kaplan–Meier overall survival curves stratified by chemotherapy treatment (chemo vs non‐chemo) in low and high Nectin‐4 expression groups in the institutional Essen cohort (A) and external validation Lund validation cohort (B). [file BJU-136-297-s001.docx]

**Spatial Distribution and Subtype-Specific Patterns of Nectin-4 Expression in Muscle-Invasive Bladder Cancer**

*Csilla Olah, Lara Sichward, Boris Hadaschik, Christopher Darr, Viktor Grünwald, Ulrich Krafft, Barbara T. Grünwald, Osama Mahmoud, Mulham Al-Nader, Peter Nyirady, Henning Reis, Tibor Szarvas*

**Supplementary Material**

**Figures**

**
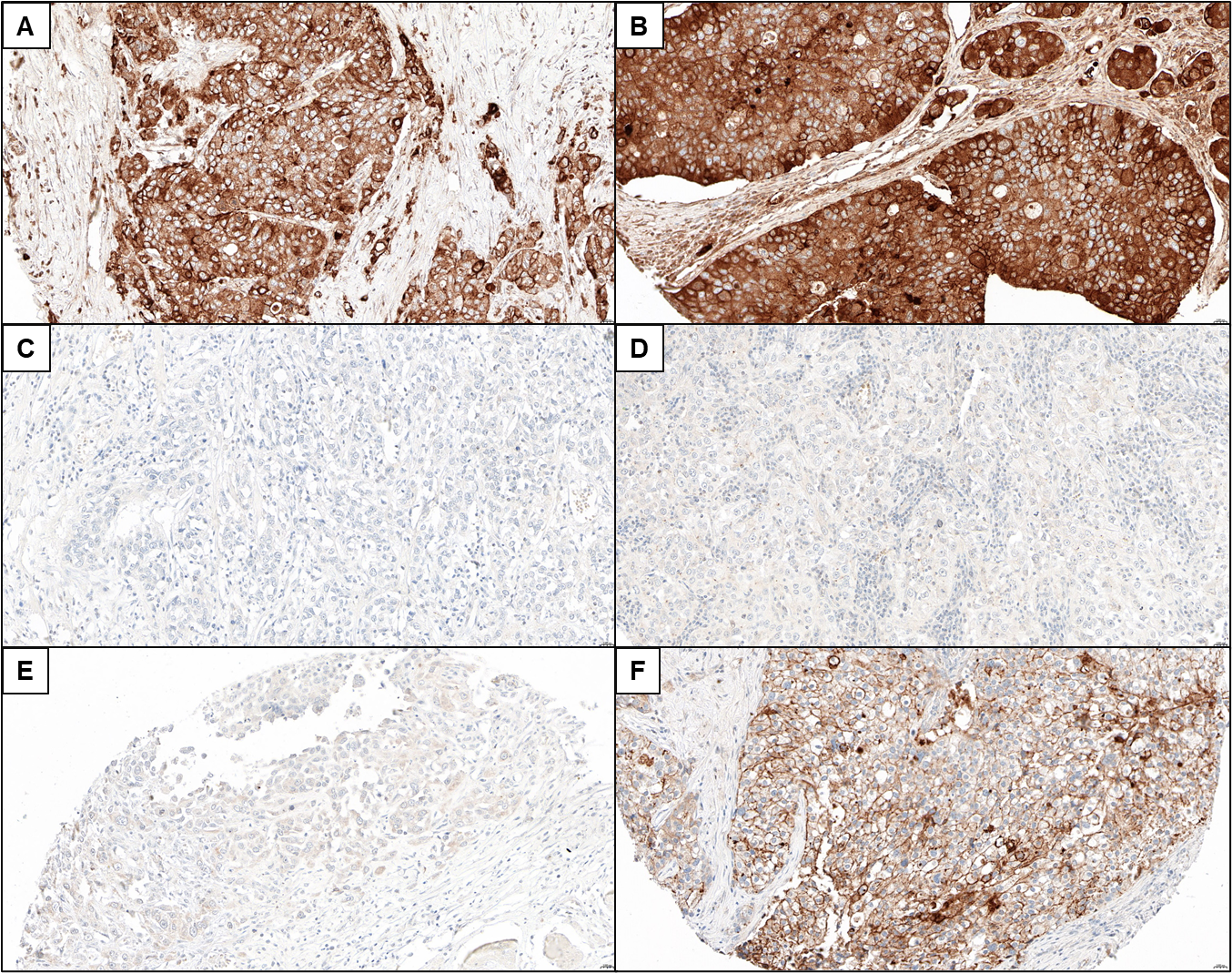
**

**Supplementary Figure 1.** Histomicrophotographs of different scenarios in Nectin-4 membranous staining. Representative primary tumor tissues from the central region (TC) and corresponding LN metastases were depicted. Positive Nectin-4 staining both in the primary tumor (A) and LN (B) are shown in the upper plots. Absence of Nectin-4 staining both in the primary tumor (C) and LN (D) are shown in the middle plots. Lastly, a case with negative TC (E) and positive LN (F) is shown in the bottom line. Of note, the constellation of positive Nectin-4 expression in the primary tumor but no expression in the LNs were rarely occurring. All images are taken at 400x magnification.


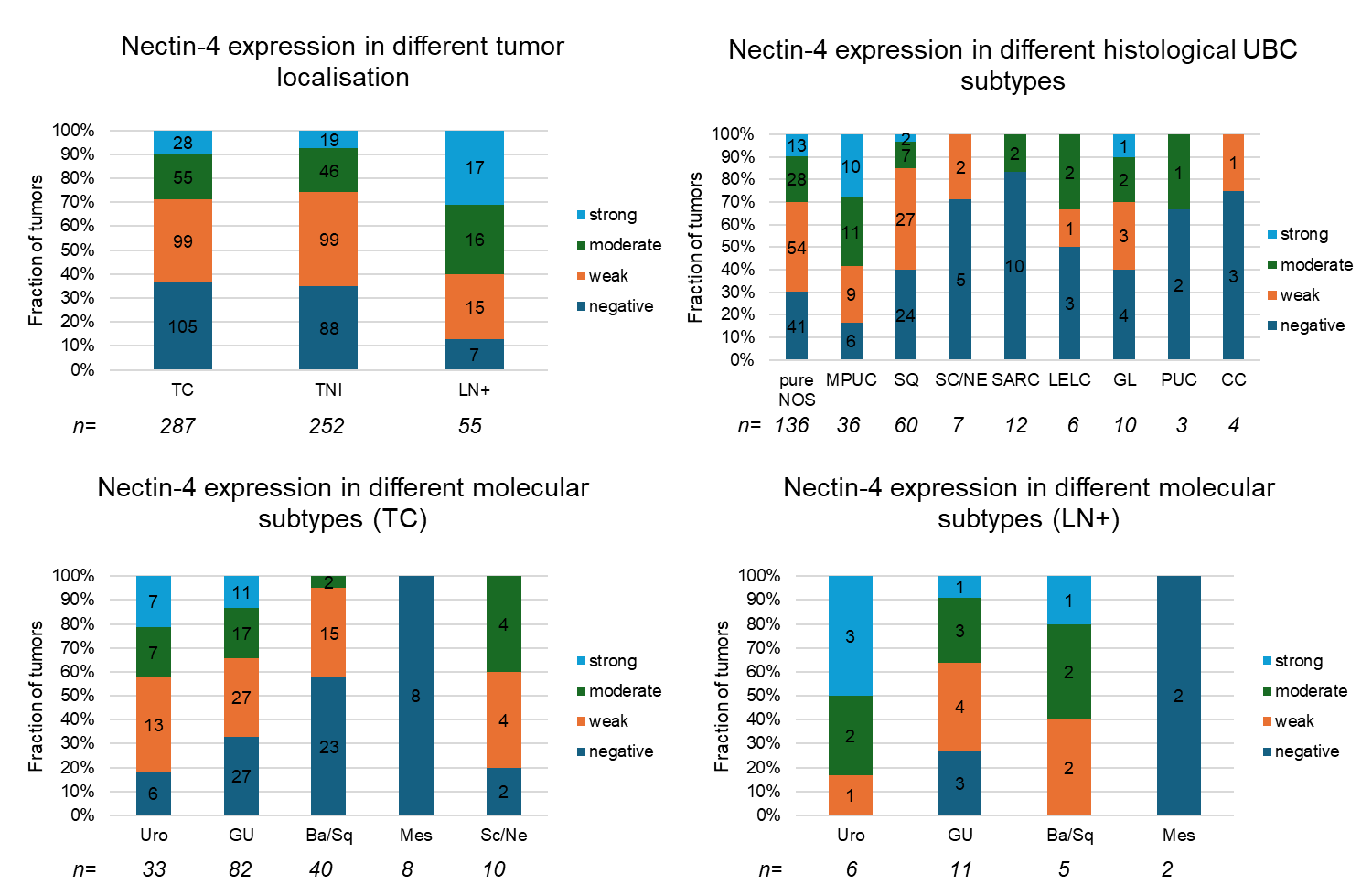


**Supplementary Figure 2.** Nectin-4 expression in different localization and in primary tumors with different histological subtypes. Nectin-4 expression in primary tumors and positive lymph nodes with different molecular subtypes. TC: tumor central, TNI: tumor-normal interface, LN+: positive lymph node, NOS: urothelial carcinoma without subtype / not otherwise specified, MPUC: micropapillary urothelial carcinoma, SQ: squamous urothelial carcinoma, SC/NE: small-cell/neuroendocrine, LELC: lymphoepithelioma-like, SARC: sarcomatoid, GL: glandular-cell, PUC: plasmacytoid, CC: clear cell, Uro: urothelial-like, GU: gemonically unstable, Ba/Sq: basal/squamous, Mes: mesenchymal-like, Sc/Ne: small-cell/neuroendocrine-like.


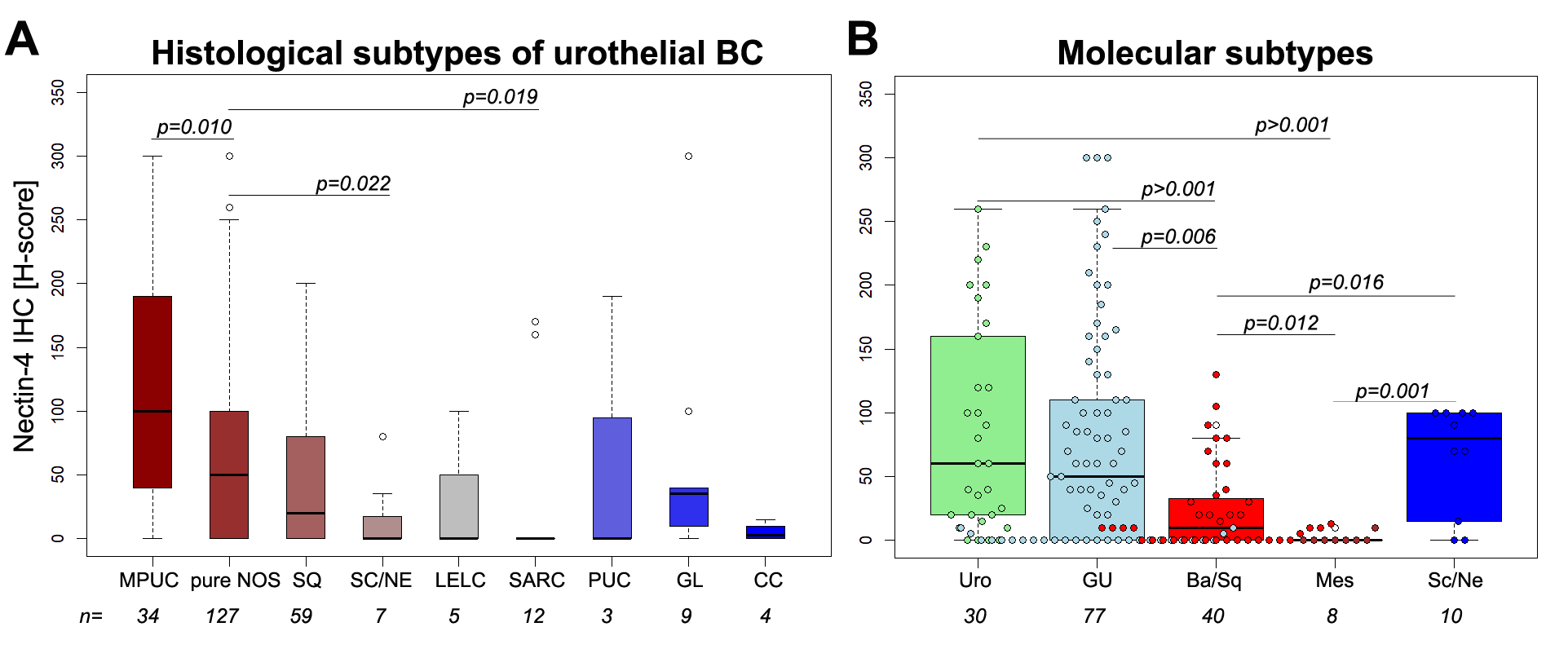


**Supplementary Figure 3.** Nectin-4 expression in muscle-invasive bladder tumors across different histological subtypes (A) and molecular subtypes (B) in the tumor central region. NOS: urothelial carcinoma without subtype / not otherwise specified, MPUC: micropapillary urothelial carcinoma, SQ: squamous urothelial carcinoma, SC/NE: small-cell/neuroendocrine, LELC: lymphoepithelioma-like, SARC: sarcomatoid, GL: glandular, PUC: plasmacytoid, CC: clear cell, Uro: urothelial-like, GU: genomically unstable, Ba/Sq: basal/squamous, Mes: mesenchymal-like.


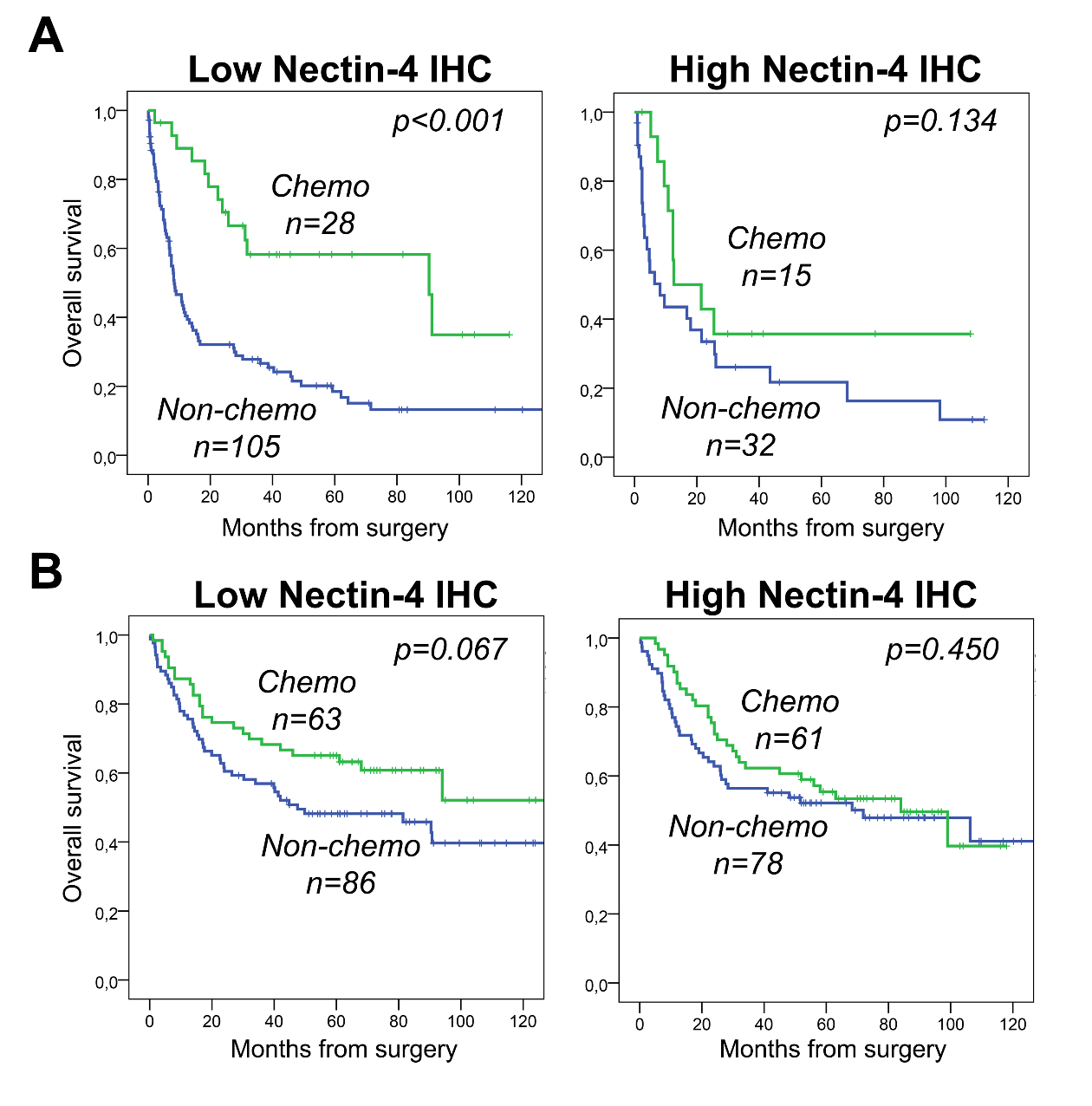


**Supplementary Figure 4.** Kaplan–Meier overall survival curves stratified by chemotherapy treatment (chemo vs. non-chemo) in low and high Nectin-4 expression groups in the institutional Essen cohort (A) and external validation Lund validation cohort (B).

**Tables**

**Supplementary Table 1.** Chi2 correlation between Nectin-4 expression and patients’ clinicopathological parameters in tumor central regions and positive lymph nodes**.**

|  | **Tumor central** | | | |  |  | **positive LNs** | | | |  |
| --- | --- | --- | --- | --- | --- | --- | --- | --- | --- | --- | --- |
| **Nectin-4 expression** | low (≤1) | | high (>1) | |  |  | low (≤1) | | high (>1) | |  |
| **Variables** | n | % | n | % | P |  | n | % | n | % | P |
| Age |  |  |  |  |  |  |  |  |  |  |  |
| ≤ 65 | 55 | 58 | 40 | 42 | 0.584 |  | 4 | 24 | 13 | 76 | 0.428 |
| > 65 | 117 | 61 | 74 | 39 |  |  | 13 | 34 | 25 | 66 |  |
| Sex |  |  |  |  |  |  |  |  |  |  |  |
| Male | 130 | 59 | 91 | 41 | 0.484 |  | 13 | 33 | 27 | 68 | 0.677 |
| Female | 42 | 64 | 24 | 36 |  |  | 4 | 27 | 11 | 73 |  |
| Stage |  |  |  |  |  |  |  |  |  |  |  |
| T1 -pT2 | 47 | 52 | 43 | 48 | 0.057 |  | 2 | 25 | 6 | 75 | 0.669 |
| pT3 -pT4 | 115 | 64 | 64 | 36 |  |  | 15 | 33 | 31 | 67 |  |
| Positive surgical margin |  |  |  |  |  |  |  |  |  |  |  |
| no | 135 | 60 | 90 | 40 | 0.371 |  | 15 | 38 | 25 | 63 | 0.296 |
| yes | 31 | 63 | 18 | 37 |  |  | 2 | 20 | 8 | 80 |  |
| Vascular invasion |  |  |  |  |  |  |  |  |  |  |  |
| no | 137 | 60 | 92 | 40 | 0.899 |  | 11 | 28 | 28 | 72 | 0.403 |
| yes | 31 | 61 | 20 | 39 |  |  | 6 | 40 | 9 | 60 |  |
| Lymphovascular invasion |  |  |  |  |  |  |  |  |  |  |  |
| no | 101 | 62 | 63 | 38 | 0.417 |  | 8 | 67 | 4 | 33 | **0.003** |
| yes | 67 | 57 | 51 | 43 |  |  | 9 | 21 | 33 | 79 |  |
| Lymph node |  |  |  |  |  |  |  |  |  |  |  |
| LN0/LNx | 118 | 62 | 71 | 38 | 0.176 |  | - |  | - |  | - |
| LN+ | 44 | 54 | 38 | 46 |  |  | 17 | 32 | 36 | 68 |  |
| Distant metastasis |  |  |  |  |  |  |  |  |  |  |  |
| M0/Mx | 120 | 63 | 70 | 37 | 0.723 |  | 13 | 32 | 28 | 68 | 0.452 |
| M+ | 10 | 59 | 7 | 41 |  |  | 1 | 17 | 5 | 83 |  |

**Supplementary Table 2.** Nectin-4 expression in different secondary histological subtype groups. NOS: urothelial carcinoma without subtype / not otherwise specified, MPUC: micropapillary urothelial carcinoma, SQ: squamous urothelial carcinoma, SC/NE: small-cell/neuroendocrine, LELC: lymphoepithelioma-like, SARC: sarcomatoid, GL: glandular-cell, PUC: plasmacytoid, CC: clear cell.

|  | **pure NOS** | **MPUC** | **SQ** | **SC/NE** | **SARC** | **LELC** | **GL** | **PUC** | **CC** |
| --- | --- | --- | --- | --- | --- | --- | --- | --- | --- |
| Number of cases | 136 | 36 | 60 | 7 | 12 | 6 | 10 | 3 | 4 |
| **Nectin-4 IHC** |  |  |  |  |  |  |  |  |  |
| negative (%) | 41 (30) | 6 (17) | 24 (40) | 5 (71) | 12 (83) | 3 (50) | 4 (40) | 2 (67) | 3 (75) |
| weak (%) | 54 (40) | 9 (25) | 27 (45) | 2 (29) | 0 | 1 (17) | 3 (30) | 0 | 1 (25) |
| moderate (%) | 28 (20) | 11 (31) | 7 (12) | 0 | 2 (17) | 2 (33) | 2 (20) | 1 (33) | 0 |
| strong (%) | 13 (10) | 10 (28) | 2 (3) | 0 | 0 | 0 | 1 (10) | 0 | 0 |
| **Nectin-4 exp.** |  |  |  |  |  |  |  |  |  |
| low (%) | 95 (70) | 15 (42) | 51 (85) | 7 (100) | 10 (83) | 4 (67) | 7 (70) | 2 (67) | 4 (100) |
| high (%) | 41 (30) | 21 (58) | 9 (15) | 0 | 2 (17) | 2 (33) | 3 (30) | 1 (33) | 0 |

**Supplementary Table 3.** Nectin-4 expression in different molecular subtype groups. LN: lymph node, Uro: urothelial-like, GU: gemonically unstable, Ba/Sq: basal/squamous, Mes: mesenchymal-like, Sc/Ne: small-cell/neuroendocrine-like,

|  | **Tumor central** | | | | |  | **positive LNs** | | | | |
| --- | --- | --- | --- | --- | --- | --- | --- | --- | --- | --- | --- |
|  | **Uro** | **GU** | **Ba/Sq** | **Mes** | **Sc/Ne** |  | **Uro** | **GU** | **Ba/Sq** | **Mes** | **Sc/Ne** |
| Number of cases | 33 | 82 | 40 | 8 | 10 |  | 6 | 11 | 5 | 2 | 0 |
| **Nectin-4 IHC** |  |  |  |  |  |  |  |  |  |  |  |
| negative (%) | 6 (18) | 27 (33) | 23 (58) | 8 (100) | 2 (20) |  | 0 | 3 (27) | 0 | 2 (100) | 0 |
| weak (%) | 13 (39) | 27 (33) | 15 (38) | 0 | 4 (40) |  | 1 (17) | 4 (36) | 2 (40) | 0 | 0 |
| moderate (%) | 7 (21) | 17 (21) | 2 (5) | 0 | 4 (40) |  | 2 (33) | 3 (27) | 2 (40) | 0 | 0 |
| strong (%) | 7 (21) | 11 (13) | 0 | 0 | 0 |  | 3 (50) | 1 (9) | 1 (20) | 0 | 0 |
| **Nectin-4 exp.** |  |  |  |  |  |  |  |  |  |  |  |
| low (%) | 19 (58) | 54 (66) | 38 (95) | 8 (100) | 6 (60) |  | 1 (17) | 7 (64) | 2 (40) | 2 (100) | 0 |
| high (%) | 14 (42) | 28 (34) | 2 (5) | 0 | 4 (40) |  | 5 (83) | 4 (36) | 3 (60) | 0 | 0 |
